# Antioxidant Potential of Selected Korean Edible Plant Extracts

**DOI:** 10.1155/2017/7695605

**Published:** 2017-11-06

**Authors:** Yaejin Woo, Hyeji Lee, Yong-Seob Jeong, Ga Young Shin, Ja Gyeong Oh, Jong-Sang Kim, Jisun Oh

**Affiliations:** ^1^School of Food Science and Biotechnology, Kyungpook National University, Daegu 41566, Republic of Korea; ^2^Department of Food Science and Technology, Chonbuk National University, Jeonju 54896, Republic of Korea

## Abstract

This study aimed to evaluate the antioxidant activity of various plant extracts. A total of 94 kinds of edible plant extracts obtained from the Korea Plant Extract Bank were screened for cytotoxicity, following which the total phenolic content of 24 shortlisted extracts was determined. Of these, extracts from three plants, namely,* Castanea crenata* (CC) leaf,* Camellia japonica* (CJ) fruit, and* Viburnum dilatatum* (VD) leaf, were examined for antioxidant capabilities by measuring radical scavenging activity, ferric reducing/antioxidant power, and lipid peroxidation inhibitory activity. In addition, cellular antioxidant activities of the three extracts were assessed by a cell-based dichlorofluorescein assay and antioxidant response element (ARE) reporter activity assay. The results demonstrated that all three extracts concentration-dependently scavenged free radicals, inhibited lipid peroxidation, reduced the cellular level of reactive oxygen species, and increased ARE-luciferase activity, indicating antioxidant enzyme-inducing potential. In particular, CJ extract showed significantly greater antioxidative activity and antimigratory effect in a breast cancer cell line compared to CC and VD extracts. Hence, CJ extract deserves further study for its* in vivo* functionality or biologically active constituents.

## 1. Introduction

Oxidative stress caused by free radicals and their derivatives leads to disturbances in redox homeostasis [1]. Reactive oxygen species (ROS) are endogenously produced during intracellular metabolic processes but can also be generated by exogenous stimuli such as UV radiation, pollutants, smoke, and drugs [2, 3]. When intracellular oxidative status increases, the cell triggers its defense systems or undergoes apoptosis [2]. These responses to oxidative stress influence numerous cellular processes including core signaling pathways, which are associated with development of systematic and/or chronic disorders including aging and cancer [3, 4]. Therefore, it is critical to remove cellular oxidants and restore redox balance.

Edible plants or plant-derived compounds can be used medicinally as preventive and/or therapeutic measures against a variety of disorders [5]. A number of studies attribute health benefits of dietary plants to biologically active constituents that possess strong antioxidant activity [6]. This has resulted in extensive studies on plant extracts or constituents that are capable of scavenging free radicals and inhibiting lipid peroxidation [7, 8].

It has been well established that the antioxidant capacity of plant extracts is closely associated with their total phenolic content. Furthermore, some antioxidants are known to activate cellular antioxidant defense systems [9, 10]. The nuclear factor erythroid 2- (NFE2-) related factor 2 (Nrf2), a transcription factor, is known to be a master regulator of the cellular antioxidant response. This has given rise to the strategy of searching for substances inducing Nrf2 activation in development of functional foods or nutraceuticals [1, 9].

In the present study, we screened 94 edible plant extracts for cytotoxicity and chose 24 of these for measurement of total phenolic contents. In addition, we tested antioxidant activity, performed antioxidant response element (ARE) reporter assays for three selected plant extracts, and examined their antimigratory effects in a human breast cancer cell line, MCF-7.

## 2. Materials and Methods

### 2.1. Preparation of Plant Extracts

A total of 94 edible plant extracts were purchased from the Korea Plant Extract Bank (Ochang, Chungbuk, Korea) [11]. According to the supplier, each plant material was washed, air-dried at 70°C, ground, and extracted in 100% methanol at 30°C for 3 days. The extract solution was then filtered and vacuum-evaporated to dryness. For* in vitro* measurement of antioxidant activity, the obtained extracts were dissolved in dimethyl sulfoxide (DMSO; Sigma-Aldrich, St. Louis, MO, USA) at 20 mg/mL.

### 2.2. Determination of Total Phenolic Contents

The total phenolic contents of the extracts were measured as described by Ariffin and coworkers [12, 13] with minor modifications [13], using a high-performance liquid chromatography system equipped with a diode array detection module (HPLC-DAD; Waters Corp., Milford, MA).

### 2.3. Determination of Cytotoxicity

To test the cytotoxicity of each extract, Cell Counting Kit-8 (CCK-8; Dojindo Laboratories, Kumamoto, Japan) was used as previously described [14]. Cytotoxicity of each extract was expressed as an IC_50_ value that indicates the concentration of the extract at which cell viability is reduced by 50% in comparison to the control treatment. The IC_50_ values were obtained by nonlinear regression using GraphPad Prism (ver. 3.0).

### 2.4. Determination of Free Radical Scavenging Capacity

Antioxidant activity of the extracts was assessed by the ability to scavenge 2,2-diphenyl-1-picrylhydrazyl (DPPH) or 3-ethylbenzothiazoline-6-sulfonic acid (ABTS) radicals as previously described [15]. Butylated hydroxytoluene (BHT), a synthetic antioxidant, or *α*-tocopherol was used as a positive control for both assays.

### 2.5. Determination of Ferric Reducing/Antioxidant Power (FRAP)

The FRAP assay was performed as previously described [16, 17]. *α*-Tocopherol was used as a positive control at concentrations of 50, 100, 500, and 1,000 *μ*M in comparison to the negative control (a solvent-treated condition).

### 2.6. Measurement of Lipid Peroxidation Inhibition

The supernatant of mouse liver homogenate was used for thiobarbituric acid reactive substances (TBARS) assay measuring the level of an end-product of lipid peroxidation, malondialdehyde (MDA) [17]. After various concentrations of samples or positive control BHT were mixed with the liver homogenate, peroxidation was induced using 20 mM ferric chloride. The absorbance of the reactant was measured at 532 nm. The lipid peroxidation inhibitory activity of a sample was calculated.

### 2.7. Cell Culture

The human breast cancer cell line MCF-7, used for the cytotoxicity assay, was obtained from the Korean Cell Line Bank (KCLB, Seoul, Korea) and maintained in Dulbecco's modified Eagle's medium (DMEM) supplemented with 10% fetal bovine serum (FBS) and 1% penicillin-streptomycin (all from Invitrogen, Carlsbad, CA, USA).

A human hepatoma cell line HepG2, obtained from KCLB, was transfected with pGL4.37[*luc2P*/ARE/Hygro] vector (Promega, Madison, WI, USA) as previously described [18]. The transfectant carrying an ARE-luciferase construct was named HepG2-ARE and cultured in the maintenance medium including 0.4 mM hygromycin (Sigma-Aldrich). All cultures were kept in a culture incubator (37°C, 5% CO_2_, humidified) for the designated period.

### 2.8. Quantification of Cellular Oxidative Stress: Dichlorofluorescein (DCF) Assay

The intracellular reactive oxygen species (ROS) concentration was quantified by measuring the oxidation level of 2,7-dichlorodihydrofluorescein diacetate (DCFH-DA; Sigma-Aldrich) as described by Wang and Joseph [19]. MCF-7 cells were treated with samples at designated concentrations in 0.5% FBS-containing culture medium for 24 h. ROS production was induced by 100 *μ*M* tert*-butyl hydroperoxide (tBHP), an oxidant, for 4 h before termination of sample treatment. The cells were then treated with 50 *μ*M DCFH-DA for 1 h at 37°C. After removal of the excess DCFH-DA, fluorescence was measured using a microplate reader at excitation and emission wavelengths of 485 and 535 nm.

### 2.9. Measurement of Antioxidant Response Element (ARE) Activity Assay

Luciferase reporter assay was conducted on HepG2-ARE cells as described [18, 20]. The cells were treated with samples for 12 h after serum starvation (0.5% FBS, 12 h). The luciferase activity, which corresponded to the ARE activity, was measured using a luciferase assay system (Promega) according to the manufacturer's instruction. Sulforaphane (Sigma-Aldrich), an isothiocyanate, was used as an ARE activator. Brusatol (Carbosynth Ltd., Newbury, Berkshire, UK), a quassinoid, was used as a specific inhibitor of the Nrf2 pathway [21]. The luminescence of the assay was detected and calibrated on total protein amounts. The data were then normalized against the control values.

### 2.10. Cell Cycle Analysis

To determine the proliferative capacity of cultured cells, 5-ethynyl-2′-deoxyuridine (EdU) uptake analysis was performed using Click-iT® EdU flow cytometry assay kit (Life Technologies). For the assay, cells were prepared as recommended by the manufacturer's instruction. Briefly, cells were cultured for 48 h and subsequently treated with 10 *μ*M EdU for 2 h, harvested, and washed in phosphate-buffered saline (PBS; Gibco) containing 1% bovine serum albumin (BSA; Sigma-Aldrich). After fixation and permeabilization, EdU-incorporation was visualized in Click-iT reaction cocktail containing Alexa Flour® 488 azide. After being rinsed, 1 × 10^4^ cells per condition were analyzed by the BD FACSCalibur flow cytometer (BD Biosciences).

### 2.11. Cell Migration Assay

For measurement of* in vitro* cell migration [22], MCF-7 cells were plated onto a 6-well plate coated with 10 *μ*g/mL of poly-L-ornithine (Sigma-Aldrich) and 5 *μ*g/mL of human plasma fibronectin (Life Technologies) at a density of 1 × 10^5^ cells per well. At about 90% confluence in the growth medium (DMEM containing 10% FBS), an artificial gap was created on a cell monolayer by scraping the cells in a straight line with a P200 pipet tip. After removing the detached cells, the growth medium was replaced with 2% FBS-containing medium for the designated period in the absence or presence of phorbol ester (12-*O*-tetradecanoylphorbol-13-acetate; TPA), an enhancer of cell motility [23, 24]. Culture images were captured at the beginning and every 24 h for the designated period using an optimal microscope (Labomed TCM 400, Labo America, Inc., Fremont, CA, USA, photographed by Eyecam, Bimeince, Suwon, Korea). The migration rate was calculated as follows: migration rate (%) = [(width at 0 h − width at 24 h)/width at 0 h] × 100.

### 2.12. Statistical Analysis

The obtained data were analyzed by one-way analysis of variance and Duncan's multiple range test using the SPSS statistics 22 software (SPSS Inc., Chicago, IL, USA). Comparisons between two groups were performed by Student's unpaired* t*-test, and *p* values less than 0.05 were considered significant. Statistical differences were indicated with asterisks, hashtags, or different alphabetical letters.

## 3. Results and Discussion

A total of 94 plant extracts were screened for cytotoxicity (partially shown in Table 1) and a selection of them were tested for total phenolic content. Cytotoxicity was assessed based on MCF-7 cell viability at various concentrations of each extract and expressed as IC_50_ values. According to the screening program of the National Cancer Institute, USA, a plant extract is generally considered actively cytotoxic if the IC_50_ value is ≤20 *μ*g/mL [25, 26]. As the IC_50_ values of all sample extracts tested in the study were higher than 20 *μ*g/mL, the test samples could be considered not actively cytotoxic. To maximize the probability of antioxidant activity expression and ensure the nontoxicity of the test samples, extracts with IC_50_ values in the range of 20 to 200 *μ*g/mL were selected (Table 1). The 24 selected extracts were then analyzed for total phenolic content by HPLC analysis. The extracts from fruits of* Camellia japonica* (CJ), leaves of* Viburnum dilatatum* (VD), and leaves of* Castanea crenata* (CC) showed the highest values for total phenolic content (Table 1) and were therefore subjected to further antioxidant assays.

**Figure 1:**
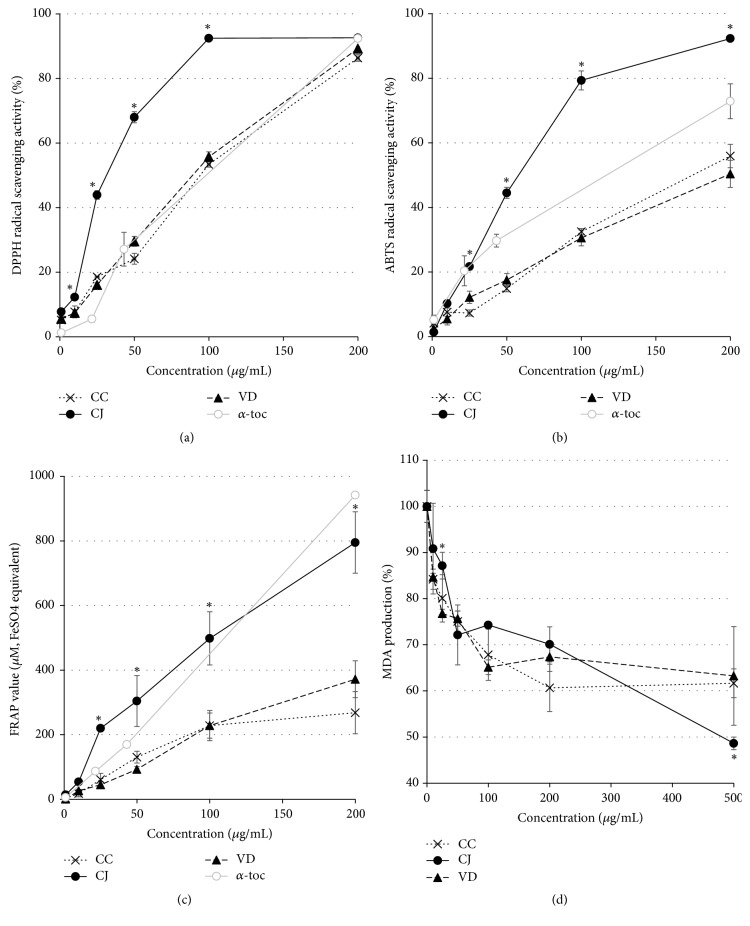
Antioxidant capabilities of CC, CJ, and VD extracts. The three extracts were examined for DPPH radical scavenging activity (a), ABTS radical scavenging activity (b), and FRAP (c) at various concentrations (1, 10, 25, 50, 100, and 200 *μ*g/mL). *α*-Toc, *α*-tocopherol, is a positive control. Lipid peroxidation inhibitory activity (d) was measured at 1, 10, 25, 50, 100, 200, and 500 *μ*g/mL.* N* (number of independent experimental sessions) = 3; error bars, mean ± SEM. Statistical differences were indicated with asterisks for comparisons between two groups, CJ and CC or VD, at the given concentration.

The antioxidant capabilities of these extracts were evaluated by measurement of radical scavenging activity, FRAP assay, and lipid peroxidation inhibition testing (Figure 1). All three kinds of extracts induced an increase in radical scavenging activity and FRAP values in a concentration-dependent manner (Figures 1(a)–1(c)). In particular, the scavenging activity and FRAP values of CJ extract were significantly greater than those of VD or CC extracts at concentrations ≥ 25 *μ*g/mL. In addition, lipid peroxidation inhibitory activity was determined via measurement of MDA levels using mouse liver tissue homogenate treated with various concentrations of the extracts (Figure 1(d)). The concentration-dependent decrease in MDA levels observed was consistent with radical scavenging activities of the extracts. These results demonstrated that CC, CJ, and VD extracts have relatively strong antioxidant capabilities.

**Figure 2:**
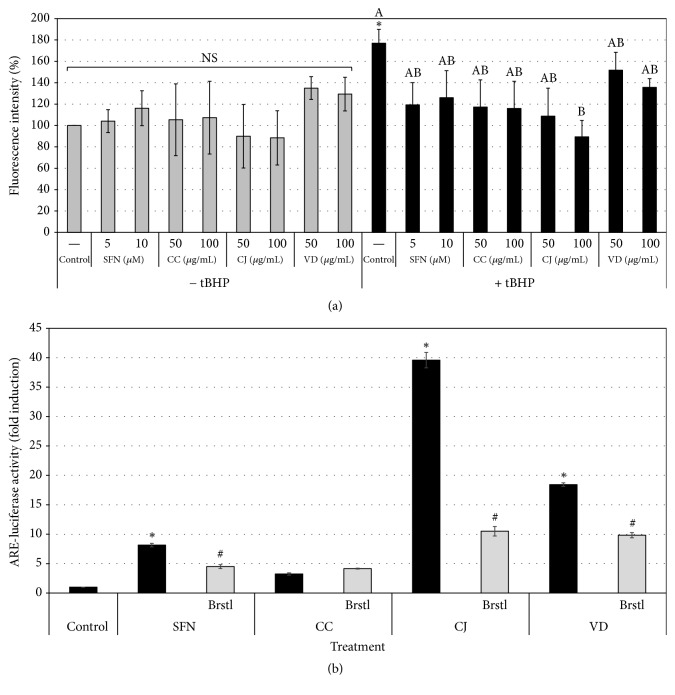
Cellular antioxidant effects of CC, CJ, and VD extracts. (a) MCF-7 cells were treated with the designated extracts and then exposed to tBHP. The intracellular ROS levels were determined by DCF fluorescence intensity. Treatment with CJ extract at 100 *μ*g/mL effectively reduced the tBHP-induced ROS levels. *N* = 3; error bars, mean ± SEM. Asterisk indicates a significant difference in comparison with the control (no tBHP treatment). Different alphabetical letters indicate significant differences among the tBHP-treated conditions. (b) HepG2-ARE cells were treated with the extracts (100 *μ*g/mL) and ARE activities were assayed. SFN, sulforaphane (5 *μ*M), is an ARE activator. Brstl, brusatol, is an Nrf2 inhibitor. *N* = 3; error bars, mean ± SEM. Asterisks indicate significant differences in comparison with the control (no treatment). Hashtags for the brusatol-treated conditions indicate significant differences in comparison with their counteracting conditions, in which cells were treated with sample but not with brusatol.

To examine whether these extracts can protect mammalian cells from oxidative stress, cultures of a human mammary gland-derived epithelial cell line MCF-7 were treated with each extract prior to challenging them with tBHP. The intracellular ROS production was determined by the relative intensity of DCF fluorescence (Figure 2(a)). While intracellular ROS formation was significantly promoted by tBHP treatment, the augmented ROS level was significantly lowered by treatment with CC, CJ, or VD extracts. This finding is indicative of the antioxidative capability of the three sample extracts in a living cell model as well as in an* in vitro* system.

To elucidate the potential mechanism by which these extracts exert antioxidant activity, ARE-luciferase activity was measured in HepG2-ARE cells following extract treatment (Figure 2(b)). As expected, ARE-luciferase activity was significantly increased by sulforaphane, a known Nrf2 activator, and suppressed by simultaneous treatment with brusatol, an Nrf2 inhibitor. All three sample extracts were found to induce ARE activation, which was partially or completely abolished by brusatol. However, CC-induced ARE activation was not influenced by brusatol. These results suggest that the antioxidative function of CJ and VD extracts was, at least in part, mediated via the Nrf2 signaling pathway. Moreover, our previous data demonstrated that CC itself did not increase the levels of Nrf2 or its downstream gene transcripts in MCF-7 cells [18]. Considering that brusatol reversibly enhances ubiquitination and degradation of Nrf2 [21], CC-induced ARE activation, which is unaffected by brusatol, may be accomplished by different mechanism(s) from the other samples. For instance, CC may activate the Nrf2 signaling pathway through the modulation of PI3K and/or MAPK instead of directly interacting with Keap1 protein, which is present in the form of a heterodimer with Nrf2 in the cytoplasm [27–29]. However, the precise molecular mechanisms for the antioxidative function of CC extract must be revealed through further study.

**Figure 3:**
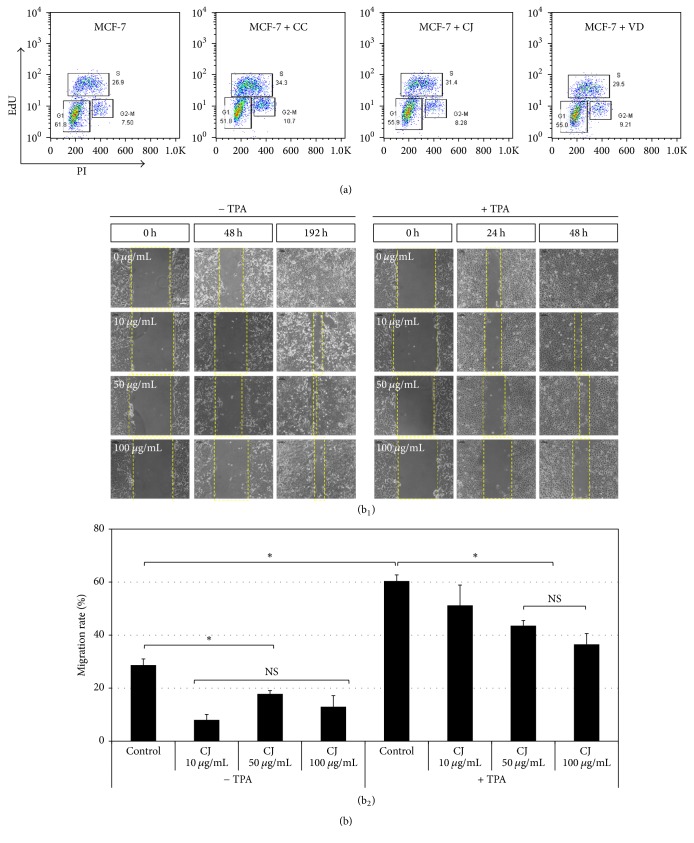
Influence of CJ extract on cell cycle progression and motility of MCF-7 cells. (a) Cells that underwent G1, S, and G2-M phases of cell cycle were analyzed by a thymidine analogue, EdU/PI-based cell sorting after treatment with each extract at a concentration of 50 *μ*g/mL for 48 h. EdU, 5-ethynyl-2′-deoxyuridine. PI, propidium iodide. (b) Cell migration assay. (b_1_) Representative images of cultured cells in the presence of CJ extract at the concentrations of 0, 10, 50, and 100 *μ*g/mL. The scale bar in a panel of (b_1_) represents 100 *μ*m, applicable to all panels in (b_1_). (b_2_) Quantification of cell migration rate under the designated cultured conditions. *N* = 3; error bars, mean ± SEM. Asterisks indicate significant differences among the conditions. NS, no significant difference.

Since Nrf2-mediated redox control is highly correlated with cancer cell proliferation [30, 31], we further examined whether those extracts influence cell cycle progression of cancer cells. MCF-7 cells that underwent G1, S, and G2-M phases were analyzed by a thymidine analogue, EdU-based cell sorting after treatment with each extract at a concentration of 50 *μ*g/mL for 48 h (Figure 3(a)). Our data showed that 61.9 ± 0.1% of cells were analyzed at G1 phase, 27.4 ± 0.5% at S phase, and 8.7 ± 1.2% at G2-M phase in the control condition. After exposure to each extract, the cells at G1 phase were slightly decreased and those at S and G2-M phases were marginally increased (data not shown). These findings demonstrate that the three extracts may not significantly influence cell cycle progression of MCF-7 cells at a dose of treatment in this study.

Intriguingly, we found that CJ extract was capable of preventing MCF-7 cell migration* in vitro* (Figure 3(b)), whereas the other two extracts were not (data not shown). Regardless of the presence of TPA, the migration rate of MCF-7 cells was decreased by treatment with CJ extract at the concentrations of ≥50 *μ*g/mL. Considering that Nrf2 can promote breast cancer cell migration which is associated with tumor aggressiveness* in vivo* [32], it is conceivable that CJ extract may contain diverse substances that work in a combinatorial manner to enhance Nrf2-mediated antioxidation potential and to reduce cancer cell motility. Identification of bioactive substances included in CJ extract and their biological functions awaits further study.

Multiple studies have demonstrated antioxidant activities of CC, CJ, and VD extracts. Several bioactive components from different parts of CJ have been reported: triterpenes in its flowers [33], flavonol glycoside in its leaves [34, 35], and saponins in its seeds [36]. Recently, it was found that the ethanol extract of CJ fruits exhibited a vascular protective effect by endothelial-dependent vasorelaxation [37] and that oleanane triterpenoids isolated from CJ fruits may be beneficial in the treatment of type 2 diabetes and obesity via PTP1B inhibitory activity [38]. Such findings suggest that CJ fruits may have biological effects through the functioning of bioavailable constituents. In addition, Noh and colleagues reported that the methanol extract of the inner shell of CC could protect hepatic cells from oxidative stress through the activation of antioxidant enzymes* in vitro* and* in vivo* and that the extract constituents scoparone and scopoletin were identified as potently active compounds [39, 40]. Kim and colleagues reported that the crude extract of VD (squeezed fruit juice) had strong antioxidant activities, reducing oxidative insults* in vitro* and* in vivo*, and that the key components involved were anthocyanins and phenolics [41, 42].

In this study, edible plant extracts were evaluated for their antioxidant potential using various* in vitro* assays. Our findings can be summarized in the form of three main contributions. Firstly, we found that CC, CJ, and VD extracts had strong free radical scavenging and lipid peroxidation inhibitory activities. Secondly, the extracts reduced tBHP-induced ROS levels, which were mediated through the activation of the Nrf2 signaling pathway. Thirdly, CJ extract among the three extracts barely affected cancer cell proliferation but decreased* in vitro* cancer cell migration. These findings demonstrated the antioxidant capability of CC, CJ, and VD extracts and potent anticancer effect of CJ, which could have implication in development of anticancer functional foods and natural source-derived nutraceuticals for cancer prevention.

## Figures and Tables

**Table 1:** List of edible plant extracts used in the study.

Serial number	Scientific name (binomial nomenclature)	Common name	Family	Part(s) extracted from^*∗*^	Total phenolic content (mg GAE/g DW)^†^	IC_50_ (*μ*g/mL)^#^
16	*Stewartia pseudocamellia*	*Stewartia koreana*	Theaceae	L	218.71 ± 11.35	119 ± 7
**18**	***Camellia japonica***	*Camellia*	Theaceae	F	561.27 ± 10.47	126 ± 2
19	*Alangium platanifolium*	*Alangium*	Alangiaceae	L	46.79 ± 1.15	104 ± 15
20	*Pseudosasa japonica*	Arrow bamboo	Gramineae	L	95.63 ± 3.84	184 ± 13
**25**	***Viburnum dilatatum***	Linden viburnum	Caprifoliaceae	L	262.56 ± 8.32	139 ± 16
27	*Eleutherococcus divaricatus *var.* chiisanensis*	*Acanthopanax chiisanensis*	Araliaceae	S	133.71 ± 4.80	182 ± 16
29	*Artemisia annua *Linne	Sweet wormwood	Compositae	L	150.06 ± 7.50	181 ± 64
32	*Zanthoxylum piperitum*	Japanese pepper	Rutaceae	L	58.71 ± 0.88	176 ± 15
33	*Anthriscus sylvestris*	Cow parsley	Umbelliferae	L, S	5.44 ± 1.45	146 ± 29
34	*Celtis sinensis*	Chinese hackberry	Ulmaceae	L	182.56 ± 8.66	109 ± 58
39	*Toona sinensis*	Chinese cedar	Meliaceae	L	135.06 ± 4.04	188 ± 22
46	*Poncirus trifoliata*	Hardy orange	Rutaceae	F	29.29 ± 2.60	158 ± 30
48	*Euonymus hamiltonianus*	Hamilton's spindletree	Celastraceae	L	52.37 ± 5.78	53 ± 2
52	*Pinus koraiensis*	Korean nut pine	Pinaceae	L	142.37 ± 7.83	181 ± 16
59	*Michelia compressa*	*Magnolia compressa*	Magnoliaceae	L	63.90 ± 2.33	107 ± 25
**61**	***Castanea crenata***	Chestnut	Fagaceae	L	256.98 ± 5.69	149 ± 46
63	*Akebia quinata*	Five-leaf Akebia	Lardizabalaceae	W	35.83 ± 2.64	37 ± 11
64	*Albizia julibrissin*	Silk tree	Leguminosae	L	129.48 ± 5.36	166 ± 80
68	*Philadelphus schrenkii*	Mock orange	Saxifragaceae	L, S	85.63 ± 3.71	129 ± 57
71	*Ipomoea batatas*	Sweet potato	Convolvulaceae	L, S	62.37 ± 0.58	55 ± 11
78	*Camellia sinensis*	Tea plant	Theaceae	L, S	256.02 ± 5.84	171 ± 47
79	*Tilia amurensis*	Amur linden	Tiliaceae	L	121.02 ± 5.92	115 ± 16
86	*Ziziphus jujuba*	Jujube	Rhamnaceae	L	244.29 ± 7.17	105 ± 33
94	*Pueraria montana*	Kudzu	Leguminosae	L	54.48 ± 0.33	97 ± 19

^*∗*^W, whole plant; F, fruit; L, leaf; S, stem; R, root. ^†^Data are expressed as milligrams (mg) of gallic acid equivalents (GAE) per 1 g dry weight (DW). ^#^IC_50_ value, the half-maximal inhibitory concentration, of cell viability was measured in a human breast cancer cell line, MCF-7; values are presented in mean ± SEM from three independent experimental sessions.

## Abbreviations

ABTS: 3-Ethylbenzothiazoline-6-sulfonic acid
ARE: Antioxidant response element
BHT: Butylated hydroxytoluene
CC: * Castanea crenata* (leaf)
CJ: * Camellia japonica* (fruit)
DCF: Dichlorofluorescein
DCFH-DA: 2,7-Dichlorodihydrofluorescein diacetate
DPPH: 2,2-Diphenyl-1-picrylhydrazyl
EdU: 5-Ethynyl-2′-deoxyuridine
FBS: Fetal bovine serum
FRAP: Ferric reducing/antioxidant power
HPLC-DAD: High-performance liquid chromatography-diode array detection
MDA: Malondialdehyde
Nrf2: Nuclear factor erythroid 2-related factor 2
PI: Propidium iodide
ROS: Reactive oxygen species
TBARS: Thiobarbituric acid reactive substances
tBHP: Tert-butyl hydroperoxide
TPA: 12-*O*-Tetradecanoylphorbol-13-acetate
VD: * Viburnum dilatatum* (leaf).
